# Application of double filtration plasmapheresis to cynomolgus monkeys: surgical techniques and a pilot study of pig fetal kidney transplantation

**DOI:** 10.1590/acb405125

**Published:** 2025-07-18

**Authors:** Genki Watanabe, Maiko Horikawa, Izumi Yamamoto, Tomohisa Endo, Takashi Watanabe, Akinori Hiratsuka, Tsuyoshi Takamura, Kenji Matsui, Naoto Matsumoto, Yatsumu Saito, Hiroshi Sasaki, Akihiko Kiyoshi, Takao Kuroda, Makoto Inoue, Eiji Kobayashi, Takashi Yokoo

**Affiliations:** 1 The Jikei University School of Medicine – Department of Clinical Engineer – Tokyo – Japan.; 2 The Jikei University School of Medicine – Division of Nephrology and Hypertension – Department of Internal Medicine – Tokyo – Japan.; 3 The Jikei University School of Medicine – Department of Urology – Tokyo – Japan.; 4 Sumitomo Pharma Co. Ltd. – Osaka – Japan.; 5 The Jikei University School of Medicine – Department of Kidney Regenerative Medicine – Tokyo – Japan.

**Keywords:** Transplantation, Heterologous, Plasmapheresis, Macaca fascicularis

## Abstract

**Purpose:**

To detail a technique to implant a double-lumen catheter to remove anti-pig antibodies. We transplanted fetal pig kidneys into cynomolgus monkeys using a double filtration plasma exchange (DFPP) protocol.

**Methods:**

Two approaches for double-lumen catheter insertion in monkeys (3–9.2 kg) were developed. DFPP was performed using hydroxyethyl starch (6%) as a replacement fluid. We transplanted fetal porcine kidneys, administered immunosuppressive agents, and evaluated the grafts.

**Results:**

The catheter insertion site was large, with postoperative hemostasis similar to blind subcutaneous puncture. Monkeys tolerated DFPP well, maintaining stable blood pressure. The technique reduced anti-pig antibodies by 67%, though acute rejection was not fully suppressed.

**Conclusion:**

A safe technique for double-lumen catheter placement in cynomolgus monkeys was developed, along with a DFPP protocol for reducing anti-pig antibodies.

## Introduction

The number of patients with chronic kidney disease is increasing worldwide, such that the shortage of donor kidneys for transplantation has become a serious problem^1^. Xenotransplantation offers a means of resolving the organ donor shortage^2^, and in recent years it has been attempted to use humanized porcine kidneys for transplantation into primates3 and even humans^4^. Some of these efforts have resulted in long-term survival, achieved using genetically modified donor organs that induce immune tolerance in combination with strong immunosuppressive therapy^4^–^6^. Our group developed a method allowing the regeneration of human kidneys derived from human-induced pluripotent stem cells, in which fetal kidneys from animals such as pigs serve as scaffolds^7^–^10^. Compared to mature vascularized organs, fetal organs are less immunogenic and can be engrafted with less immunosuppression^11^,^12^.

The clinical success of kidney xenotransplantation requires a determination of whether a viable kidney xenograft can be obtained with the same level of immunosuppression as currently used in human kidney transplantation. However, previous studies have demonstrated the need to remove preexisting donor-specific antibodies by plasma exchange for the transplant to be viable^13^. It has also been noted that naturally occurring anti-pig antibodies may result in rejection^14^,^15^. In clinical practice, double filtration plasmapheresis (DFPP) is used to remove donor-specific antibodies. Blood access in large animal models is well established^16^,^17^, but not yet in small animals^18^–^20^. In this study, we followed the human kidney transplantation protocol, by performing DFPP, and then evaluated its effects on fetal pig kidney transplantation to a cynomolgus monkey.

## Methods

### Experimental animals

The pigs used in this experiment are described in a previous report12. Specifically, experiments on neonatal pig kidney were performed at the laboratory of IVTec, Inc. Approval was obtained from the Animal Experimentation Ethics Committee of IVTec, Inc. (approval nos. IVT20–26 and 20–84; experiment nos. k-20–019 and k-20–051). Transplantations of neonatal or fetal pig kidneys into recipient cynomolgus monkeys were performed in the laboratories of Sumitomo Pharma Co. Ltd. (approval nos. AN12843 and AN13772). The cynomolgus monkeys used in the experiments weighed between 3 and 9.2 kg, and their use was approved by the Institutional Animal Care and Use Committee of Sumitomo Pharma Co. Ltd. All heterologous regenerative kidney experiments were approved by the Animal Ethics Committee of the Jikei University School of Medicine, Tokyo, Japan (approval no. 2020–055).

### Animal management

Recipient cynomolgus monkeys were managed as already reported12. Specifically, body temperature, body weight, and oxygen saturation were measured between 8:30 a.m. and 10 a.m., before drug administration. The animals’ general condition, including appearance, behavior, feeding, feces, and other findings, was recorded at least once a day. Blood samples were typically collected on days -20, -15, -12, -7, -4, and -1 prior to transplantation and tested for the following: red blood cells, hemoglobin (Hb), hematocrit (Hct), mean corpuscular volume, mean corpuscular hemoglobin, mean corpuscular hemoglobin concentration, reticulocytes, white blood cells, neutrophil, lymphocyte, mononuclear cell, eosinophil, basophils, platelets, prothrombin time, activated partial thromboplastin time, fibrinogen (Fbg), total protein, albumin, total bilirubin, aspartate aminotransferase, alanine aminotransferase, alkaline phosphatase, gamma-glutamyl transpeptidase, blood urea nitrogen (BUN), creatinine (Cr), sodium (Na), potassium (K), chlorine (Cl), calcium, phosphorus, and glucose (Glu), total cholesterol, triglycerides, and C-reactive protein. Electrolyte changes by DFPP were measured using i-STAT (Abbott, United States of America) for Na, K, Cl, ionized calcium, total carbon dioxide concentration, Glu, BUN, and Cr. Hct, Hb, anion gap, and activated clotting time (ACT) were measured using Hemocron Jr. Signature+ (Heiwa Bussan, Japan). Measurements of serum concentrations of drugs started on day -7. After transplantation, blood samples were obtained twice a week, with additional samples drawn as needed according to the results of the previous tests.

### Double lumen catheter insertion procedure

#### Cervical approach

**Figure 1:**
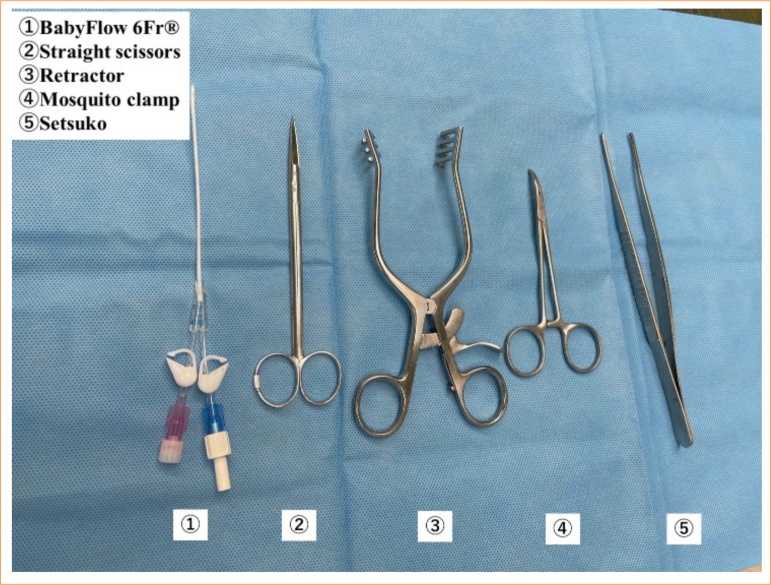
The instruments used in the surgery. Double-lumen catheter for dialysis, syringe for local anesthesia, Surflow (Terumo, Japan), guidewire, scissors, mosquito-peanut, forceps, wound openers, and gauze.

Source: Elaborated by the authors.

**Figure 2:**
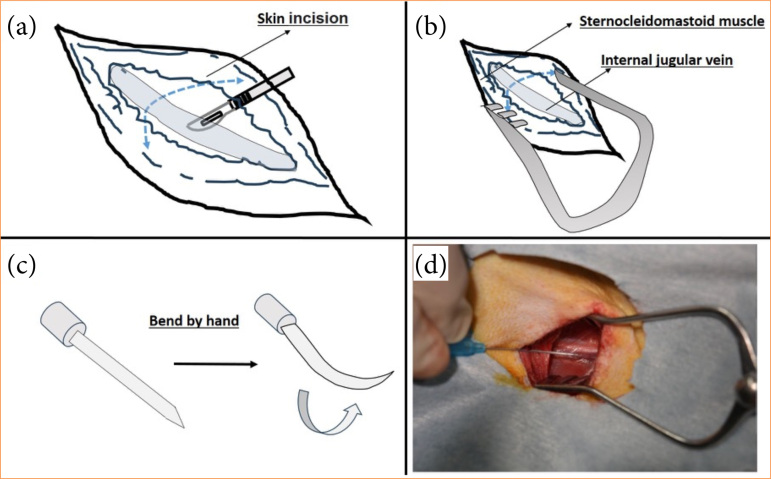
The schema of cervical approach.

Source: Elaborated by the authors.

**Figure 3:**
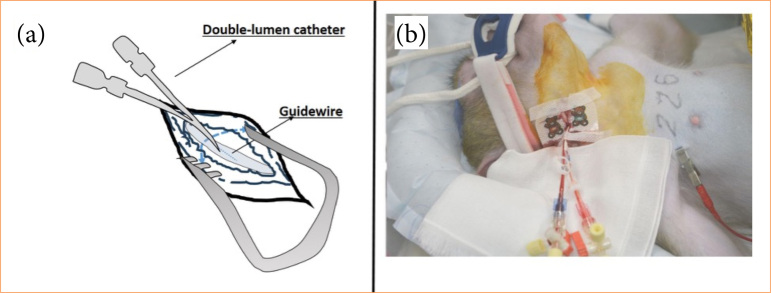
Fixation of double-lumen catheters at the neck.

Source: Elaborated by the authors.

The surgical instruments Surflow (Terumo, Japan), for test puncture, guide wire, and the double lumen catheter, were prepared on the sterile drape (Fig. 1). A skin incision of approximately 8 cm in length was made along the right cervical region (Fig. 2a). The superficial muscles were vertically dissected, and the sternocleidomastoid muscle was displaced outward to expose the internal jugular vein (Fig. 2b). Using a surface needle, the internal jugular vein was punctured from the body surface, and to prevent the needle from penetrating the vein, the outer cannula was carefully advanced, with a Surflow (Terumo, Japan) puncture needle bent manually as needed depending on the depth (Fig. 2c). The exposed internal jugular vein was then punctured using this curved needle, and a guide wire was inserted after removing the inner cannula (Fig. 2d). The double lumen catheter was filled with heparinized saline, and using the guide wire, it was inserted into the internal jugular vein and secured at a depth of approximately 10 cm (Fig. 3a). The catheter was then fixed to the skin using 5-0 silk sutures, and the skin was partially sutured and further secured with Tegaderm (3M, United States of America) (Fig. 3b). After the completion of plasma exchange, the double lumen catheter was removed.

**Figure 4:**
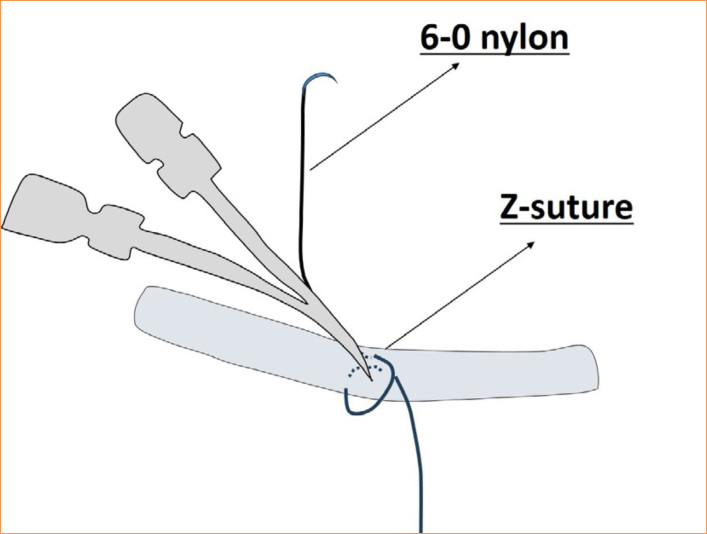
Illustration of double-lumen catheter fixation.

Source: Elaborated by the authors.

Due to anticoagulation measures during the procedure, such as heparin administration, precautions were taken to prevent postoperative bleeding. Specifically, the double lumen catheter scheduled for removal was lifted with the left hand, and a 6-0 nylon suture was inserted along the catheter at the insertion site. Knots were tied both below and above the catheter, and a Z-shaped suture was added before removing the catheter, ensuring complete hemostasis (Fig. 4).

#### Inguinal approach

**Figure 5:**
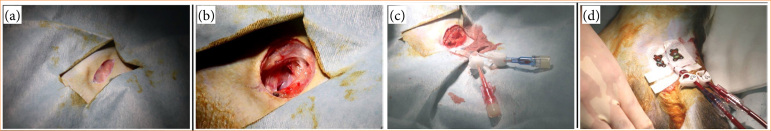
The schema of inguinal approach.

Source: Elaborated by the authors.

The left inguinal region was used. After thorough disinfection of the surgical site with iodine, a sterile drape was applied. First, a skin incision of approximately 4 cm was made along the inguinal ligament (Fig. 5a). The femoral vein and femoral artery were visualized under direct vision while moving toward the head (Fig. 5b). A small skin incision was made below the skin incision site, and Surflow (Terumo, Japan) was inserted. After confirming backflow, the inner cannula needle was removed, and the guide wire was inserted. A dilator was introduced, and after tunneling through the skin, the double lumen catheter was inserted (Fig. 5c). The catheter was secured on the skin (Fig. 5d). After the completion of plasma exchange, the double lumen catheter was removed, and compression hemostasis was applied. However, due to anticoagulation measures during the procedure, such as heparin administration, the subcutaneous tunnel area was sutured with 4-0 sutures in a knot fashion to ensure complete hemostasis.

#### Measurement of anti-pig mononuclear cell antibodies

A flowchart explaining the measurement of anti-pig mononuclear cell antibodies is provided in Suppl. Fig.1^21^. Antibodies titers were measured before and after DFPP using flow cytometry. Cynomolgus monkey serum was collected as follows: blood was added to serum separation microtubes and left at room temperature for ~30 min, then centrifuged (15°C, 5,000 rpm, 5–10 min) in a small cooling centrifuge (CF15R, Himac, Japan). Anti-pig mononuclear cell antibodies in whole blood were measured as follows: diluting whole pig blood 2-fold with phosphate buffered saline (PBS), with Ficoll-Paque Premium 1.084 (Cytiva, Japan). The diluted blood was gently added on top of the liquid layer of Ficoll-Paque and then centrifuged at 400 g for 30 min, after which the monocyte fraction was collected with a pipette. Then 10-mL PBS was added, followed by centrifugation at 300 g for 5 min and collection of the supernatant. This washing process was repeated. Then 1-mL hemolytic reagent was reacted with the sample for 3 min, followed by two rounds of the above washing procedure. The monocytes were counted and then diluted with PBS to adjust the cell concentration to 1 × 10⁵ cells/mL. Stored cynomolgus monkey serum was diluted 20 times with PBS and added to the pig monocyte solution at the ratio of 1:1 and allowed to react with the cells at 4°C for 30 min. Then, 1-mL PBS was added, followed by centrifugation at 300 g for 5 min and removal of the supernatant. This washing process was repeated. FITC-labeled goat anti-monkey IgG antibody diluted 200 times with PBS was added as a secondary antibody, and the reaction was allowed to proceed at 4°C for 30 min under light-shielding. Then, 1-mL PBS was added, followed by centrifugation at 300 g for 5 min and removal of the supernatant. This washing process was repeated. Finally, 500-µL 1% PFA was added, and anti-pig mononuclear cells antibody titers were measured on a fluorescence-activated cell sorter (MACS Quantify). The cell fraction of each sample was gated, and the median absorbance at 525 nm of the cells was measured. Using PBS as a negative control, each measured value was corrected and defined as the antibody titer.

### Double filtration plasmapheresis

The monkeys were pretreated with atropine (0.1 mg/kg), administered intramuscularly (i.m.), followed by 10-mg ketamine/kg i.m. for anesthesia induction, and 0.5–2% isoflurane inhalation for anesthesia maintenance. Intraoperatively, electrocardiogram (lead II), blood pressure (determined using a cuff), body (rectal) temperature, heart rate, and oxygen saturation were monitored using a Bioscope AM130 (Fukuda M.E. Industries). An intravenous route of administration was secured using a Surflow (Terumo, Japan) indwelling needle, infusion route, and infusion pump using Terumo Fusion Type 28 (Terumo, Japan). During surgery, the animals received a dextrose solution containing vitamins as a supplement. For analgesia, 0.1-mg butorphanol/kg was administered i.m. at the beginning and end of surgery. Benzylpenicillin potassium at the dose of 50,000 units was administered i.m. at the beginning and at the end of surgery to prevent infection.

**Figure 6:**
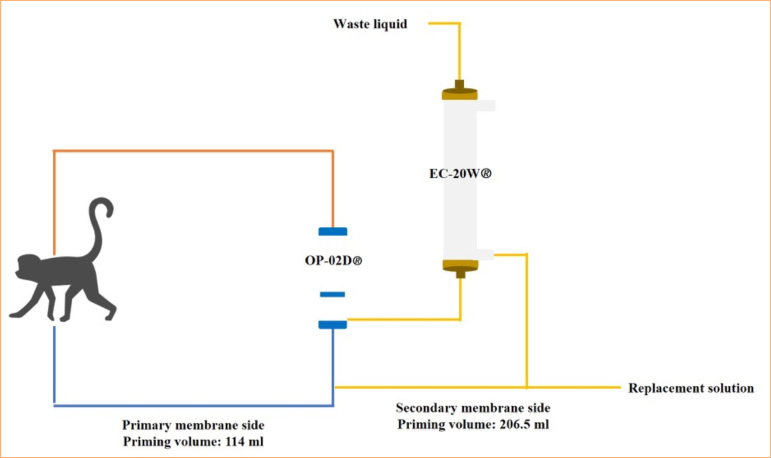
Double filtration plasmapheresis circuit diagram. The dialysis circuit consisted of the primary membrane circuit and the secondary membrane circuit. The plasma separation membrane was OP-02D and the plasma component separation membrane Cascade Flow EC-20W. The priming volume of the primary membrane side was 114 mL, and the PV of the secondary membrane side was 206.5 mL.

Source: Elaborated by the authors.


A schematic diagram of the circuit used for DFPP is shown in Fig. 6. DFPP was performed using a versatile blood purification system ACH-Σ (Asahi Kasei Medical, Japan). The dialysis circuit consisted of PE-PSG (Naniwa Rubber Industries, Japan) as the primary membrane circuit and DFPP-SGD (Naniwa Rubber Industries) as the secondary membrane circuit. The plasma separation membrane was OP-02D (Asahi Kasei Medical), and the plasma component separation membrane was Cascade Flow EC-20W (Asahi Kasei Medical). The priming volume (PV) of the primary membrane side was 54 mL for the primary membrane side circuit and 60 mL for the plasma separation membrane (25 mL blood side/35 mL plasma side), totaling 114 mL. The PV of the secondary membrane side was 56.5 mL for the secondary membrane side circuit, and 150 mL for the plasma component separation membrane, totaling 206.5 mL. The total plasma volume was calculated by Eq. 1 for the plasma treatment volume, which was 1.5 (experiment 1) and 2 (experiments 2 and 3) times the plasma volume for each experiment:

Total Plasma volume = Body weight (kg) × 0.065 × (1−hematocrit (%)/100) (1)

The blood flow rate was 6 mL/kg/min, and the plasma separation rate was 20–30% of the blood flow rate. The drain pump was set at 15% of the plasma separation rate when an increase in intra-circuit pressure was determined, with the replacement fluid pump operated at the same rate to replenish the replacement fluid. Vascular access was established via the cervical or femoral vein using a BabyFlow 6Fr (NIPRO, Japan). Unfractionated heparin (200 U) was administered as the anticoagulant at the time of priming, with the continuous dose adjusted based on the activated clotting time during treatment. Calcium gluconate hydrate (CGH, 8.5%) was administered as needed to prevent hypocalcemia caused by the sodium citrate in the blood products.

To determine the effectiveness of DFPP, immunoglobulin assay kits were used to detect IgM, IgA, and IgG. Autowaco IgG N, Autowaco IgA N, Autowaco IgM N and Immunocalibrator Set N (Fujifilm Wako Pure Chemicals Corporation) were used as reagents. These measurements were made on an automatic analyzer JCA-ZS050 (JEOL Ltd.).

### Plasma preparation

The PV and replacement fluid in the circuit were set up as follows: because the PV of the extracorporeal circuit exceeded 10% of the fluid volume, it had to be filled with blood for the primary membrane side and with plasma for the secondary membrane side. Blood was collected from the femoral vein, radial cutaneous vein, or saphenous vein of cynomolgus monkeys once a week at a maximum volume of 4.8 mL/kg/week. Plasma was prepared from another cynomolgus monkey with the same blood type. Blood was collected as follows: 5–10 mg ketamine/kg was administered i.m. under anesthesia. Blood for whole blood samples was slowly mixed with citrate-phosphate-dextrose-adenine (CPDA) at the ratio of 10:1.4, collected in a separator bag (150-mL capacity), refrigerated at 4°C, and sterilized by radiation (30 Gy) before use.

Plasma was prepared from whole blood as follows: the obtained blood was promptly mixed with ACD-A at the ratio of 10:1.5 and centrifuged at 3,000 rpm for 5 min using a Hitachi 05PR-22 (Hitachi, Japan) cooling centrifuge. The plasma was placed in a separator bag and frozen at -20 to -30°C. To prevent contamination, the blood storage operation was performed aseptically. The prepared plasma was used as PV and as replacement fluid.

In experiments 1 and 2, whole blood was used for priming the primary membrane side, and the plasma prepared from the blood of another monkey was used as the priming and replacement fluid in the secondary membrane side. However, in experiment 2, to compensate for the insufficient plasma volume, 6% hydroxyethyl starch (HES, Otsuka Pharmaceutical Co. Ltd., Japan) was added, and the plasma was used after its one-time passage through the plasma component separation membrane, to reduce the content of anti-pig mononuclear cell antibodies. In experiment 3, 6% HES was used as the priming and replacement solutions for the secondary membrane side circuit.

### Harvesting transplanted tissue

Pretreatment and anesthesia induction/maintenance were as described in the section “DFPP.” Then the entire abdomen of the monkey was disinfected with isodine, and the abdomen was opened through a median incision. After graft retrieval, the abdomen was closed using the clipped area as a guide. The recovered transplanted tissue was preserved in 4% paraformaldehyde (PEA) for pathological analysis.

### Evaluation of sample tissue

Collected specimens were fixed with 4% PFA, paraffin-embedded, sectioned into sections 4-µm thick, and then stained with hematoxylin and eosin, periodic acid-Schiff, and Masson trichrome. For the detection of CD3 antigen, formalin-fixed specimens were deparaffinized, and antigen retrieval was performed by incubating the sections in 0.01 M sodium citrate buffer (preheated for 30 s) in a pressure cooker for 10 s. Then, the sections were quickly washed with PBS. Endogenous peroxidase was blocked with 3% hydroxidase. After a subsequent PBS wash, the sections were blocked in 5% skim milk for 30 min, incubated overnighted at 4°C with anti-CD3 monoclonal antibody (MA5–12577; Invitrogen, United States of America) as primary antibody, washed with PBS, and reacted with Histofine Simple Stain (424151; Japan) for 30 min. Hematoxylin was used for counterstaining.

### Immunosuppressants

The immunosuppressive agents used for human kidney transplantation were also used for desensitization prior to kidney transplantation (Table 1, Suppl. Fig. 2^21^). Tacrolimus (TAC, 0.01–0.06 mg/kg × 2) was administered i.m. twice daily starting on day -10; mycophenolate mofetil (MMF, 25–50 mg/kg × 2) was administered orally twice daily starting on day -13 except on days of DFPP; methylprednisolone (3.33–0.67 mg/kg) was administered on day -6. Basiliximab (1 mg/kg) was administered intravenously on the day of transplantation and on day 4. Rituximab (10 mg/kg) was administered intravenously on day -16 (day -18 in experiment 3). Blood concentrations of TAC and MMF were measured seven days before transplant, four days before, on the day of transplant, and every four days thereafter with the doses adjusted based on target trough values, and these were set in advance in the protocol.

**Table 1:** List of drugs used.

Agent (company)	Dose
**Induction**	
Basiliximab (Novartis International)	1 mg/kg/day
Rituximab (Zenyaku Kogyo)	10 mg/kg/day
**Maintenance**	
FK506 (Astellas)	0.01–0.06 mg/kg × 2/day
Mycophe-nolate mofetil (Pfizer)	25–50 mg/kg × 2/day
	33.3 mg/kg × 2/day (days -7~)
Methylprednisolone (Pfizer)	0.67–3.33 mg/kg/day
	8.33 mg/kg/day (day 0)
**Adjunctive**	
Famotidine (LTL)	0.25 mg/kg × 2/day
Aspirin (Bayer)	40 mg/kg/day
Roxadustat (Astellas)	10 mg/kg/day
Valganciclovir (Mitsubishi)	15 mg/kg/day
Low molecular weight heparin (Kaken)	700 IU/body/day
Erythropoietin (Kyowa Kirin)	2,000 U/body/day
Butorphanol (Bristol Myers)	0.1 mg/kg × 2/day
Benzylpenicillin (Meiji Seika)	10万 U/body/day

Source: Elaborated by the authors.

### A pilot study of pig fetal kidney transplantation

#### Experiment 1: verification of the safety and efficacy of double filtration plasmapheresis in cynomolgus monkeys

**Figure 7:**
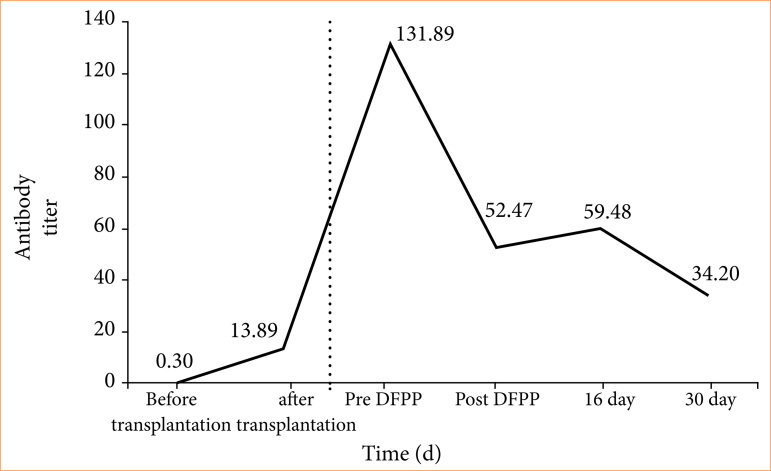
Experiment 1: double filtration plasmapheresis (DFPP) in sensitized cynomolgus monkeys and changes in anti-pig mononuclear cell antibody titers. The anti-pig mononuclear cell antibody titer was 0.30 before sensitization, whereas on day 125 post-transplantation it was 131.89. Immediately after DFPP, it was 52.47 and on days 16 and 30 post-DFPP 59.48, and 34.2, respectively, indicating that DFPP reduced the antibody titer by ~60%.

Source: Elaborated by the authors.

DFPP was performed on a cynomolgus monkey sensitized by neonatal pig kidney transplantation to test its effects on recipient safety and its ability to decrease anti-pig mononuclear cell antibody titers. Figure 7 summarizes the changes in antibody titers. DFPP was performed only once, on day 125 after sensitization by neonatal pig kidney transplantation, with anti-pig mononuclear cell antibody titers measured before and immediately after DFPP and on days 16 and 30 thereafter. The safety of the experimental animals during the study was also evaluated.

#### Experiment 2: effect of double filtration plasmapheresis with cynomolgus monkey plasma in fetal pig kidney transplantation

Two cynomolgus monkeys (5.5 and 9.2 kg) were selected. Because anti-pig mononuclear cell antibodies were present in the plasma, their removal was necessary before the plasma could be used in the experiment (Suppl. Fig. 3^21^). The desensitization protocol was equivalent to that used in human kidney transplants (Suppl. Fig. 2^21^), and DFPP was performed only once. Blood concentrations of TAC and MMF were measured twice weekly, with the doses adjusted based on target trough values (Suppl. Fig. 4: 5.5 kg; and Suppl. Fig. 5: 9.2 kg^21^).

For lowering anti-pig mononuclear cell antibody titers, the plasma treatment volume was twice the plasma volume. During DFPP, an arterial line was placed in the tail artery, and blood pressure was monitored continuously during the procedure. Phenylephrine was administered when the monkey’s blood pressure was low. To prevent hypocalcemia, a 1-mL bolus dose of 8.5% CGH was administered at the start of DFPP, followed by its continuous administration (3 mL/h). Serum levels of Ca^2+^ were monitored, and the CGH administration rate was adjusted accordingly.

On day 11 after DFPP, kidneys from fetal pigs were transplanted into the cynomolgus monkeys. Pretreatment, anesthesia, and the abdominal incision were as described above. A trocar with an inner diameter of 5–10 mm was inserted in the abdomen as a laparoscopy port. Insufflation of the abdominal cavity was performed, and the recipient’s cavernous and para-aortic areas were identified laparoscopically. A pocket was created for transplantation in each site, and the kidneys were then transplanted and clipped. Insufflation was terminated, the trocar was removed, and the wound was closed. On day 22 post-transplantation, the grafts were harvested for pathological evaluation.

#### Experiment 3: effect of double filtration plasmapheresis with 6% hydroxyethyl starch in fetal pig kidney transplantation

One cynomolgus monkey (7 kg) was selected. The desensitization protocol was equivalent to that used in human kidney transplants (Table 1 and Suppl. Fig. 2^21^). Two rounds of DFPP were conducted, and fetal pig kidney transplantation was performed nine days thereafter. On day 33 post-transplantation, specimens were collected for pathological evaluation. Preoperative and postoperative preparations were the same as in experiment 2, with the following changes: 6% HES was used instead of cynomolgus monkey plasma as the priming and replacement fluids on the secondary membrane side. To prevent a decrease in the levels of coagulation factors, plasma components remaining in the circuit after the end of treatment were continuously administered until the monkey awakened from anesthesia.

## Results

Before performing DFPP, a double-lumen catheter insertion was conducted in both the neck and groin regions. The respective advantages and considerations are summarized in Table 2. The double-lumen catheter, due to its larger insertion point, facilitated postoperative hemostasis by passing through subcutaneous tissues like a blind puncture. However, in cases in which the position and trajectory of the blood vessels made subcutaneous puncture challenging, a method involving concomitant skin incision was adopted to enable direct puncture of exposed veins. When puncturing blood vessels under direct visualization, it was desirable to apply our recommended Z-suture before catheter removal, followed by hemostasis through ligature at the cut end during removal. Additionally, when inserting catheters or similar instruments into narrow blood vessels, we employed a method in which the curved tip of the injection needle was used as a guide while inserting the catheter^22^. Using the above methods, double-lumen catheters were installed, and DFPP was performed in three cases of cynomolgus monkeys.

**Table 2:** The comparison of neck and groin approach.

	Merits	Pitfalls
Cervical approach	Central venous placement (passes through the mixed vein)	Slightly challenging puncture
Inguinal approach	Safe puncture	Verification of the tip location is required

Source: Elaborated by the authors.

### Experiment 1

Figure 7 summarizes the anti-pig mononuclear cell antibody titers over the course of experiment 1. Before transplantation, the titer was 0.30 whereas on day 29 post-transplantation it was 13.89. An attempt was made to harvest the kidney on the same day, but the graft was lost, suggesting tissue melting due to acute rejection. DFPP was performed 125 days after sensitization. Before DFPP, the anti-pig mononuclear cell antibody titer was 131.89. The effect of DFPP was evaluated by anti-pig mononuclear cell antibody titer values: 52.47, 59.48, and 34.2 immediately after DFPP, 16 days after DFPP, and 30 days after DFPP, respectively, showing a decrease of 25.2 (57.4%). Adverse effects were delayed awakening from anesthesia, hypoglycemia, and hypocalcemia (Ca^2+^ < 0.7 mmol/L). No decrease in Fbg and no significant changes in vital signs occurred during DFPP, as assessed by electrocardiogram and measurements of temperature, heart rate, and oxygen saturation. During DFPP, blood pressure measurements were not always possible.

### Experiment 2

**Figure 8:**
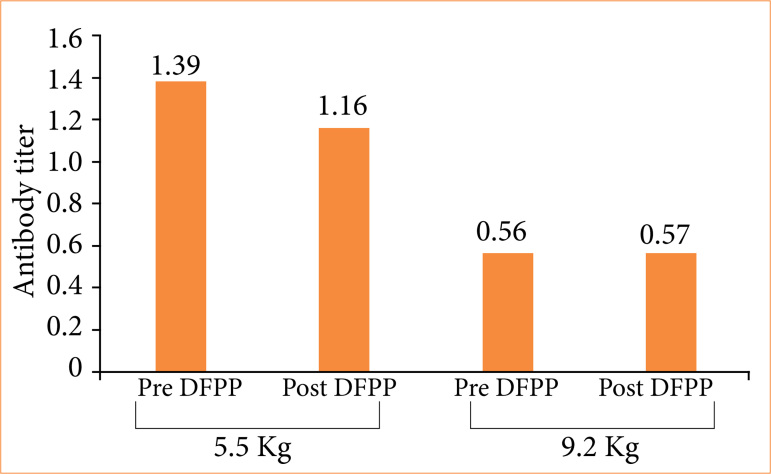
Experiment 2: removal of anti-pig mononuclear cell antibodies by double filtration plasmapheresis (DFPP) using cynomolgus monkey plasma. The anti-pig mononuclear cell antibody titers of selected cynomolgus monkeys before and immediately after DFPP were 1.39 and 0.56, respectively, and antibody titers on days 16 and 30 after DFPP were 1.16 and 0.565, respectively, a decrease of 16 and 0%.

Source: Elaborated by the authors.

**Figure 9:**
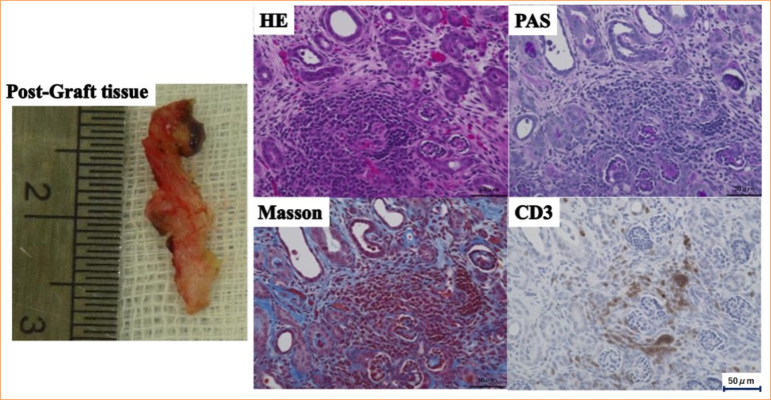
Experiment 2: development and pathological analysis of the transplanted tissue. Grafts were harvested on day 33 after one round of double filtration plasmapheresis (DFPP) (two animals). The left upper panel shows the graft tissue of pre-transplant (5 mm), and the left lower panel shows the tissue (14 mm) on day 33 post-transplant. The right panel revealed hematoxylin and eosin, Masson, PAS and CD3 staining. The edematous changes in the interstitium and a high degree of accumulation of CD3-positive lymphocytes were observed.

Source: Elaborated by the authors.

As the anti-pig mononuclear cell antibody titer in plasma collected from the monkeys was 2.7 (Suppl. Fig. 3^21^), DFPP was performed prior to the experiment to ensure a reduction to 0.465. The anti-pig mononuclear cell antibody titers of selected cynomolgus monkeys before and immediately after DFPP were 1.39 and 0.56, respectively, and antibody titers on days 16 and 30 after DFPP were 1.16 and 0.565, respectively, a decrease of 16 and 0% (Fig. 8). Grafts were harvested on day 33 after one round of DFPP (two animals). The graft tissue was 5 and 8 mm at the time of implantation, but it had grown to 14 and 20 mm by day 33 (Fig. 9). Pathological examination revealed edematous changes in the interstitium and a high degree of accumulation of CD3-positive lymphocytes (Fig. 9). In areas in which the renal tissue structure could be seen, there was a strong inflammatory cell infiltrate and tubulointerstitial fibrosis. Side effects, including hypocalcemia or a decrease in Fbg, were not observed.

### Experiment 3

**Figure 10:**
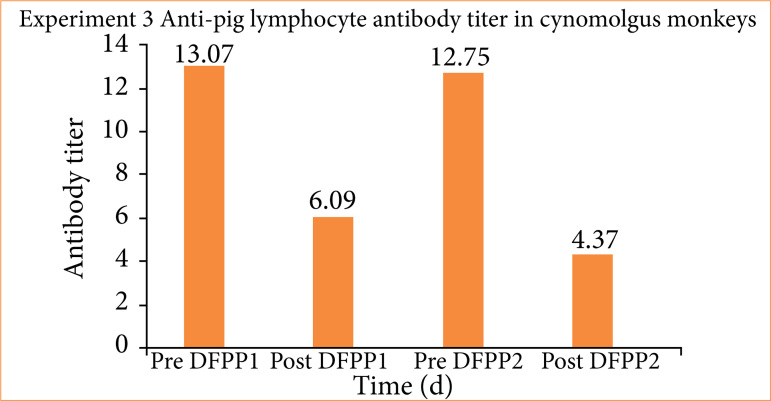
Experiment 3: removal of anti-pig mononuclear cell antibodies by double filtration plasmapheresis (DFPP) using 6% hydroxyethyl starch. The anti-pig mononuclear cell antibody titer decreased from 13 before DFPP to 6.09 after the first round of DFPP and 4.36 after the second, resulting in a 67% decrease.

Source: Elaborated by the authors.

**Figure 11:**
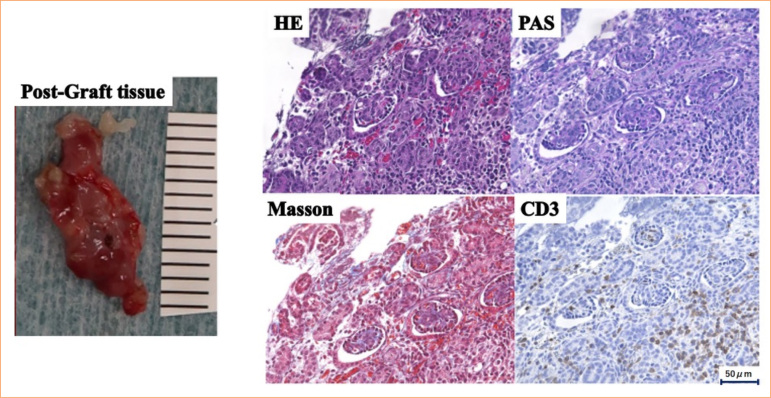
Experiment 3: development and pathological analysis of the transplanted tissue. The left panel shows the graft tissue which had grown from 6 to 15 mm by day 33. The right panel shows hematoxylin and eosin, Masson, PAS, and CD3 staining. Pathological findings revealed edematous changes in the interstitium, and the accumulation of CD3-positive lymphocytes implies that rejection were not fully suppressed.

Source: Elaborated by the authors.

**Figure 12:**
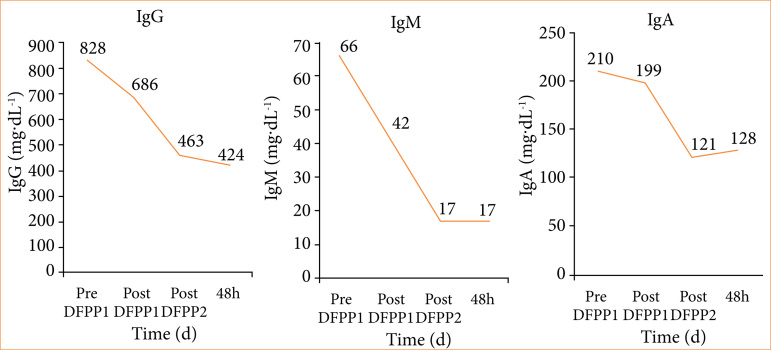
Experiment 3: changes in serum concentrations of IgG, IgM, and IgA before and after double filtration plasmapheresis (DFPP). During DFPP, the serum levels of IgG, IgM, and IgA were measured and found to be reduced from 828 to 424 mg/dL, from 66 to 17 mg/dL, and from 210 to 128 mg/dL, respectively.

Source: Elaborated by the authors.

A sufficient reduction of the anti-pig mononuclear cell antibody titer was achieved using 6% HES as the replacement solution, with DFPP performed twice. The anti-pig mononuclear cell antibody titer decreased from 13 before DFPP to 6.09 after the first round of DFPP and 4.36 after the second, resulting in a 67% decrease (Fig. 10). This was a much larger reduction than in the other experiments. The transplanted pig fetal kidneys were harvested 33 days after transplantation (Fig. 10). The graft tissue was 6 mm at the time of transplantation but had grown to 15 mm by day 33. Unfortunately, pathological examination revealed edematous changes in the interstitium, and the accumulation of CD3-positive lymphocytes implies that rejection was not fully suppressed (Fig. 11). Despite the use of 6% HES alone, there was no decrease in Fbg (Fig. 12). To confirm a sufficient reduction of anti-pig mononuclear cell antibody titers, serum levels of IgG, IgM, and IgA were measured and found to be reduced from 828 to 424 mg/dL, from 66 to 17 mg/dL, and from 210 to 128 mg/dL, respectively (Fig. 12).

## Discussion

Overcoming antibody- and T-cell-mediated rejection early in solid-organ transplantation is crucial to the realization of xenotransplantation. The use of high-dose immunosuppressive agents increases the likelihood of infection, which in immunosuppressed patients may be fatal. In xenotransplantation, two strategies may improve its practical clinical application: the use of genetically modified pigs or fetal pig kidneys, which are less immunogenic to the host, and the adoption of the same desensitization protocols used in human kidney transplants, as these are currently the most common method of desensitization in the clinical setting.

In humans, preemptive DFPP has been employed to remove anti-A and anti-B antibodies in ABO-incompatible transplants^23^, but its use in animals has not been extensively investigated^18^,^19^. Thus, the goal of this study was to prevent the rejection of a low-immunogenic fetal pig kidney by applying the desensitization protocol used in humans. In this study, we could not control rejection, but we established and evaluated methods for performing DFPP in light-weight (3–9.2 kg) cynomolgus monkeys. The efficacy of DFPP was verified, and the anti-pig mononuclear cell antibody titer was reduced by 67%, which, while not sufficient, enabled short-term graft engraftment. This is the first report showing that DFPP can be performed in animals using 6% HES as the replacement fluid.

In plasma exchange, the replacement fluid of choice is usually human albumin or fresh frozen plasma. In animals, human blood products may be used, but the potential for delayed hypersensitivity reactions to the different proteins should be considered^24^-^27^. Therefore, in this study allogeneic plasma was used to accurately assess the efficacy of DFPP. The PV in this experiment was of 320 mL, which is a large volume considering the fluid volume of a 3–9.2-kg cynomolgus monkey. Therefore, the generation and use of blood products with no immunological effects were a challenge.

Experiment 1 demonstrated that DFPP could be performed using blood from other cynomolgus monkeys. However, acquiring a sufficient reservoir of allogeneic plasma was time-consuming and effort-intensive, which limited the amount of plasma available. Moreover, the plasma contained a high titer of anti-pig mononuclear cell antibodies. Therefore, in experiment 2, prior to its use as a replacement solution, the allogenic plasma was treated with a secondary DFPP membrane to remove the antibodies. However, despite sufficiently low antibody titers (Fig. 8), rejection still occurred, thus showing that even small amounts of anti-pig mononuclear cell antibodies can produce rejection (Fig. 9). We therefore considered a fluid that did not contain anti-pig mononuclear cell antibodies and had no immunological effects.

In previous studies, 3% Varihes or Isohes was used as the replacement fluid for plasma exchange in humans. Although concerns were raised regarding complications, such as a large decrease in the levels of coagulation factors, including Fbg, and associated bleeding, the decrease in Fbg was minor, and there were few side effects^28^-^29^. Therefore, we used a 6% HES infusion solution as the priming and replacement solutions.

In experiment 3, in which DFPP was performed using 6% HES as the replacement solution, a sufficient reduction in antibody titer was achieved. The histological findings showed the improved growth and development of fetal pig kidneys, but rejection still occurred (Fig. 12). This may have occurred due to the large rebound in antibody titer after DFPP and/or to the involvement of T-cell-mediated rejection. Regarding the former, antibody titers just before the first DFPP and just before the second DFPP were almost unchanged (13.1 and 12.75, respectively; Fig. 11). In the case of human ABO-incompatible transplants, decreasing preoperative antibody titers can significantly reduce antibody-associated rejection^30^. Therefore, the additional round of DFPP likely reduced antibody titers and may have effectively suppressed rejection. For the latter, the pathology evaluation showed clusters of CD3-positive cells, suggesting T-cell-mediated rejection. In humans, T-cell-mediated rejection can occur as early as six days after kidney transplantation^31^ and was thus suspected in our experimental model. Indeed, previous studies have shown that T-cell depletion is associated with long-term viability in pig-to-monkey kidney xenografts^31^. The use of antithymocyte globulin for both induction and transduction may therefore prevent rejection.

## Conclusion

We have developed a protocol to safely remove anti-pig mononuclear cell antibodies by experimental DFPP in a very small cynomolgus monkeys. Issues such as priming, replacement fluid, and monitoring of circulatory dynamics, the coagulation system, and electrolytes were appropriately addressed, resulting in a technique that can reduce anti-pig mononuclear cell antibody titers by ~67% without serious adverse events.

## Data Availability

All data are enclosed in the manuscript or available in the article supplementary material, at https://doi.org/10.5281/zenodo.15515201.
